# What’s in a name? Mixed connective tissue disease in the UK: a survey of current clinical practice and unmet need

**DOI:** 10.1093/rap/rkag135

**Published:** 2026-10-30

**Authors:** Rosalind Benson, Liza J McCann, Laura Gatti, Clare E Pain

**Affiliations:** Department of Rheumatology, NHS University Hospitals of Liverpool Group, Liverpool, UK; Department of Women’s and Children’s Health, University of Liverpool, Liverpool, UK; Department of Paediatric Rheumatology, Alder Hey Children’s NHS Foundation Trust, Liverpool, UK; Pediatric Rheumatology Unit, ERN-ReCONNET Center, Meyer Children’s Hospital IRCCS, Florence, Italy; Department of Women’s and Children’s Health, University of Liverpool, Liverpool, UK; Department of Paediatric Rheumatology, Alder Hey Children’s NHS Foundation Trust, Liverpool, UK

Key message • MCTD/overlap diseases present significant clinical challenges, highlighting the need for standardised assessment and management guidance.

Dear Editor, It is recognised that some people with autoimmune CTDs have overlapping features of multiple rheumatic conditions. Validated clinical criteria for mixed connective tissue disease (MCTD) exist [1–4], however, the existence of MCTD as a distinct disease entity is debated in the rheumatology community. The argument focuses on whether overlapping phenotypes will eventually evolve into a recognised CTD (i.e. SSc or SLE) or if phenotypically stable patients represent a distinct disease entity [5, 6]. Although no clinical criteria endorsed by the BSR, EULAR or ACR exist, BSR and ACR guidelines acknowledge overlap and mixed CTD.

Attempts to address this diagnostic void have been undertaken [7], and despite calls for international consensus clinical practice guidelines (CPGs) from the European rheumatology community, none currently exist [8]. This results in variations in care for these phenotypically complex patients.

To better understand current practice across the UK, we conducted an anonymous electronic survey of healthcare professionals (HCPs) involved in the care of patients with MCTD/overlap disease. This was disseminated through national specialist networks and regional rheumatology mailing groups. It explored diagnostic labelling, service provision, clinical assessment, organ surveillance, access to treatment, patient support resources and perceived unmet needs. No ethical approval was required to undertake this work.

A total of 43 HCPs responded, representing at least 21 identifiable rheumatology services across secondary and tertiary care in England, Wales and Scotland. Respondents included paediatric (22/43) and adult (21/43) rheumatologists, of whom 14 also provided adolescent care, and 3 allied health professionals.

Most [36/43 (83.7%)] indicated that patients seen at their centre would receive a diagnosis of MCTD. Of note, 26/43 (60.5%) considered MCTD to be a distinct disease entity whereas 5/43 (11.6%) did not, and a significant 27.9% were uncertain. Many clinicians preferred to use phenotype-led terminology or ‘overlap’ disease. There was recognition that this patient group may have an evolving disease course, developing over time a specific CTD phenotype. To avoid premature categorisation, some respondents preferred the terminology undifferentiated CTD. Other clinicians reported inconsistent use of MCTD with patients not always meeting clinical criteria, particularly during childhood.

Service delivery models demonstrated substantial variation. Patients with MCTD were seen within general rheumatology clinics at 12/43 (30%) centres, while most patients were reviewed within specialist CTD clinics. Only two adult centres reported dedicated MCTD or overlap CTD clinics. In many centres, patients were managed within disease-specific clinics such as SSc, lupus or myositis. A total of 34/43 (79%) reported all consultants delivered care to the breadth of CTD patients, whereas 9/43 (21%) reported consultants delivered specific clinics only (e.g. lupus but not SSc). Access to multispecialty clinics was available to the majority [36/43 (84%)] in order of frequency: respiratory, renal, dermatology, neurology, gastroenterology, oral medicine and cardiology.

Multidisciplinary care for these patients was patchy. Good access to specialist CTD nurses [41/43 (95.3%)] and physiotherapists or occupational therapists [39/43 (91%)] was reported. Psychology support was not universal, although it was better served in paediatric than adult settings [17/22 (77%) *vs* 7/21 (33%)].

A lack of CPGs was perceived by 25/43 (58.1%) as an unmet need, while others felt extrapolation from disease-specific guidelines was sufficient. However, our survey demonstrated variations in practice both in relation to cardiopulmonary screening and nailfold assessment (Table 1).

**Table 1:** Nailfold capillaroscopy and cardiopulmonary assessment practices.

Diagnostic screening	Survey responses for screening, *n* (%)			Additional details
	Performed in all patients	Phenotype or symptom dependent	Do not perform	
Cardiopulmonary screening	30/43 (70)	13/43 (30)	0/43 (0)	Presence of SSc features (including nailfold changes) would prompt cardiopulmonary screening by some clinicians
Nailfold capillaroscopy at presentation	9/43 (21)	16/43 (37)	18 (42)	Using dermatoscope/ophthalmoscope [22/43 (51%)] Access to USB microscopy or video capillaroscopy [11/43 (26%)]

There are multiple disease activity scores used in rheumatology that require training for correct usage. There is no composite score for use in MCTD. Instead, clinicians typically assess based on phenotype. Only 5/43 (12%) respondents felt confident in undertaking all disease scores [Manual Muscle Testing 8 (MMT8), SLEDAI, modified Rodnan skin score (mRSS) and the Clinical European League Against Rheumatism Sjögren’s Syndrome Disease Activity Index (ClinESSDAI)]. Fewer than half were confident in undertaking the mRSS. Confidence in the SLEDAI/BILAG and MMT8 was higher [31/43 (72%) and 34/43 (79%), respectively]. The heterogeneity of MCTD presents clinical challenges for accurate disease assessment.

Respondents highlighted important challenges relating to treatment access. More than half felt that an MCTD diagnosis could restrict access to high-cost therapies (e.g. rituximab, belimumab, bosentan), with some reporting a need to reclassify patients under alternative CTD diagnoses to meet commissioning requirements.

The majority (91%) of respondents identified significant unmet needs for this patient group. Common themes included lack of registry data, insufficient evidence-based treatment recommendations, limited access to clinical trials and inconsistency in diagnostic terminology. Educational resources for patients were also reported to be scarce—only 6/43 (14%) pointed to MCTD-specific leaflets, others pointed to patient organisations based on predominant phenotype. This can result in patients receiving overwhelming amounts of information, of variable relevance.

Overall, our findings (albeit UK only) reveal marked challenges in the care of patients with MCTD/overlap disease. Difficulties span across diagnosis/classification, access to care and treatments, disease assessment and organ surveillance.

We have demonstrated the need for a wider conversation through a dedicated international task force with the aim of developing formal clinical practice guidelines to reduce unwarranted variations and improve care for patients living with MCTD/overlap throughout their life course.

## Data Availability

The data underlying this article will be shared upon reasonable request to the corresponding author.
